# The current evidence on surgical management for synchronous bilateral renal tumors: results from a scoping review

**DOI:** 10.1007/s00345-023-04503-y

**Published:** 2023-07-05

**Authors:** Carlo Giulioni, Martina Maggi, Giacomo Maria Pirola, Eugenio Martorana, Angelo Cormio, Jeremy Yuen-Chun Teoh, Vineet Gauhar, Andrea Benedetto Galosi, Daniele Castellani

**Affiliations:** 1 Urology Unit, Department of Urology, Azienda Ospedaliero-Universitaria Delle Marche, Polytechnic University of Marche, 71 Conca Street, 60126 Ancona, Italy; 2 Department of Maternal-Infant and Urological Sciences, Umberto I Polyclinic Hospital, Sapienza University, Rome, Italy; 3 Department of Urology, San Giuseppe Hospital, Multimedica Group, Milan, Italy; 4 Department of Urology, Nuovo Ospedale Civile Di Sassuolo, Modena, Italy; 5 Faculty of Medicine, S. H. Ho Urology Centre, Department of Surgery, The Chinese University of Hong Kong, Hong Kong, China; 6 Department of Urology, Ng Teng Fong General Hospital, Singapore, Singapore

**Keywords:** Renal cell tumor, Synchronous bilateral renal masses, Nephron-sparing surgery, Minimally invasive surgery, Same-sitting surgery

## Abstract

**Purpose:**

Synchronous bilateral renal masses (SBRM) account for a low percentage of kidney tumors, and there is no current recommendation for their management. The objective was to review evidence regarding the best surgical approach for SBRM in terms of type and timing of surgery.

**Methods:**

A broad literature search was performed on 28th January 2023 using Scopus, PubMed, and EMBASE. Only English papers dealing with adults were included. Meeting abstracts were excluded.

**Results:**

Twenty-four papers were accepted and included. SBRM behave less aggressively than metachronous tumors, and partial nephrectomy (PN) is the preferred therapeutic option to preserve renal function. Open, laparoscopic, and robot-assisted approaches were found to be similar in oncological outcomes, though robot-assisted surgery resulted in lower comorbidities. Same-sitting PN was demonstrated to be a safe approach, particularly in the robotic-assisted one. Finally, the same-siting and staged NSS were similar in preserving renal function.

**Conclusions:**

PN should be the desirable treatment for SBRM whenever feasible and if patients are fit, but surgeon expertise should also be taken into the account.

**Supplementary Information:**

The online version contains supplementary material available at 10.1007/s00345-023-04503-y.

## Introduction

Synchronous bilateral renal masses (SBRM) are uncommon [1]. Up to 5% of patients with Renal Cell Carcinoma (RCC) present with SBRM [2]. SBRM can be sporadic or occur in hereditary cancer syndromes, where their incidence increases due to genetic variation [3]. Furthermore, patients with hereditary SBRM tend to have a more aggressive biological behavior than sporadic tumors [4].

Yet, malignant and benign concordance rates of bilateral kidney tumors are reported to be 89% and 71%, respectively [5]. In an international multicenter study of 10,337 patients with RCC, no difference in comparison in terms of cancer-specific survival (CSS) between SBRM and unilateral kidney tumor was reported, with similar 10-year survival rate [6]. The management of patients with SBRM has not yet been well-defined, and international guidelines do not report evident recommendations, mostly due to limited data. The factors influencing the therapeutic decision are similar to those for solitary tumors, such as tumor characteristics, patient comorbidities, and surgeon expertise. Nevertheless, the rationale of surgery in patients presenting with SBRM surgery is to ensure oncological radicality and, at the same time, minimize the potential risks of increased perioperative surgical complications and the detriments to renal function. Therefore, considering the tendency to chronic renal disease (CKD) in patients after nephrectomy and the consequent increased risk of cardiovascular mortality [7, 8], nephron-sparing surgery (NSS) may be a valid option for SBRM to preserve renal function. However, it is still not clear which is the best approach for such patients that is to say same-sitting or staged NSS.

We aimed to perform a scoping review focusing on the outcomes of renal surgery in patients with SBRM, evaluating surgical and oncological outcomes by either comparing simultaneous and staged procedures or comparing different surgical procedures.

## Evidence acquisition

### Literature search

In this study, we performed a scoping review evaluating surgical, functional, and oncological outcomes of bilateral NSS for kidney tumors. Literature search was performed on 28th January 2023 using EMBASE, PubMed, and Scopus. The following term and Boolean operators were used: (partial nephrectomy OR nephron-sparing surgery) AND bilateral AND (renal OR kidney) AND (tumor OR cancer OR neoplasm). No date limit was imposed.

### Selection criteria

Only English papers dealing with adults were accepted. Preclinical and pediatric studies were excluded. Reviews, letters to the editor, case reports, and meeting abstracts were also excluded.

### Study screening and selection

Only studies reporting the surgical management of SBRM were considered. Our main objective was to evaluate the oncological radicality and functional outcomes of the surgical management of SBRMs, with a secondary aim of assessing its safety. The PICOS model (Patient Intervention Comparison Outcome Study type) was used to frame and respond to the clinical question; P: adult men with SBRM; I: partial or radical nephrectomy; C: comparison with patients with metachronous bilateral renal masses or who underwent different approach, type of surgery or timing; O: overall survival, cancer-specific survival, perioperative complications, postoperative renal function; S: prospective and retrospective studies.

All retrieved studies were screened by two independent authors through Covidence Systematic Review Management® (Veritas Health Innovation, Melbourne, Australia). A third author solved discrepancies. The full text of the screened papers was selected if found pertinent to the purpose of this review. This review was registered on https://osf.io/registries/ (number osf.io/hs96k).

## Evidence synthesis

### Literature screening

Literature search found 2238 papers. 712 duplicates were automatically excluded, and 1526 papers were left for screening against title and abstract. Among them, 1453 papers were further excluded, because were irrelevant to this review purpose. The remaining 73 full-text papers were screened for appropriateness. A total of 49 papers were excluded for the following reasons: 25 were meeting abstracts, 1 was a review, 16 articles were not in English, 5 had wrong outcomes, 1 had wrong indications and 1 had wrong study design. Finally, 24 papers were accepted and included [9–32]. Supplementary Fig. 1 shows the flow diagram of the literature search.

### Study characteristics

There were 21 retrospective [10–14, 16–19, 21–32] and 3 prospective studies [9, 15, 20]. Regarding surgical techniques, 3 studies used robot-assisted surgery [18, 19, 32], 2 laparoscopy [14, 30], 12 open approach [9–12, 17, 20–24, 28, 31], and the remaining 7 different surgical procedures [13, 15, 16, 25–27, 29]. There were 6 studies performing same-sitting bilateral surgeries [14, 17–19, 26, 27], 11 staged procedures [9, 10, 16, 23–25, 28–32], and the remaining ones both approaches [11–13, 15, 20–22]. Moreover, 10 studied reported the outcomes of bilateral NSS [14, 17–19, 26, 28–32] and the others a combination of NSS and radical nephrectomy (RN) [9–13, 15, 16, 20–25, 27]. Tables 1 and 2 show the single-series and comparative studies characteristics.

**Table 1:** Case-series studies concerning synchronous bilateral renal masses.

Author Years	Country	Type of study	Number of patients	Type of surgery	Approach	Timing of surgery	Follow-up	Conclusion
Grimaldi 1998 [9]	USA	Prospective	29: 19 Synchronous, 10 Metachronous	25 RN/PN, 4 PN/PN	Open	Two stages	52 months	PN is a viable option for non-familiar bilateral RCC due to its good prognosis
Black 2000 [10]	Germany	Retrospective	7	2 PN/PN, 5 RN/PN	All open	Two stages	33 months	PN for SBRM, with at least one central tumor, had an acceptable complication rate and overall survival
Blute 2000 [11]	USA	Retrospective	94	65 RN/PN 24 PN/PN 5 RN/RN	Open	66 one stage, 28 two stages	71 months	NSS guaranteed a similar OS. CSS, LRFS, and MFS to more extended surgery, with better long-term functional outcomes
Blute 2003 [12]	USA	Retrospective	44	30 RN/PN 4 RN/RN 10 PN/PN	Open	37 one stage, 7 two stages	108 months	There were no statistically significant differences in CSS and MFS in patients with SBRM and unilateral RCC
Booth 2008 [13]	USA	Retrospective	45	17 RN/PN 3 RN/RN 23 PN/PN	Open or laparoscopic	7 one stage 36 two stages	16 months	Bilateral PN guaranteed optimal renal function preservation, maintaining adequate oncological radicality
Madi 2009 [14]	USA	Retrospective	3	All PN/PN	Hand-assisted laparoscopic	One stage	51 months	One-stage hand-assisted laparoscopic PN is a safe and feasible option in exophytic small renal masses
Hu 2017 [15]	China	Prospective	32	15 PN/PN, 12 RN/PN and 5 PN/RN	Open PN and Open/Laparoscopic RN	6 PN/PN and 2 RN/PN with one stage, 5 PN/RN, 10 RN/PN 9 PN/PN with two stages	R 6–138 months	According to tumor and patient's characteristics, an appropriate surgical approach should be performed to maximize oncological radicality and renal function preservation
Woodson 2013 [16]	USA	Retrospective	11	8 RAPN/RAPN 2 RN/PN 1 RN/PN	9 Bilateral robot-assisted, 1, robot-assisted + open, 1 robot-assisted + laparoscopic	Two stages	21 months	Bilateral PN provides durable functional and oncological outcomes in patients with SBRM
Mason 2018 [17]	USA	Retrospective	76	All PN/PN	Open	One stage	38 months	Simultaneous bilateral PN for SBRM had satisfactory perioperative outcomes, both for complication rate and renal function preservation
Otoshi 2020 [18]	Japan	Retrospective	8	PN/PN	Robot-assisted	One stage	–	Simultaneous RAPN is feasible for the preservation of renal function and oncological outcomes
Gallo 2022 [19]	Italy	Retrospective	27	All PN/PN	Robot-assisted	All one stage	30 months	Bilateral robot-assisted PN was a safe and feasible procedure, with a favorable LRFS

*RN* radical nephrectomy, *PN* partial nephrectomy, *RCC* renal cell cancer, *SBRM* synchronous bilateral renal masses, *NSS* nephron-sparing surgery, *OS* overall survival, *CSS* cancer-specific survival, *LRFS* local recurrence free survival, *MFS* metastatic free survival, *R* range

**Table 2:** Comparative studies concerning synchronous bilateral renal masses

Author (Years)	Country	Type of study	Cases	Type of surgery	Approach	Stages	Control	Type of surgery	Stages	Approach	Follow-up	Final conclusion
Novick 1989 [20]	USA	Prospective	28	5 bilateral PN 23 RN/PN	Open	In most cases two stages	28 metachronous	Not reported	Not reported	Open	50 months	5-year patient survival rate is higher in SBRM than in metachronous RCC
Boorjian 2008 [21]	USA	Retrospective	147	6 bilateral RN 82 RN/PN 46 Bilateral PN	Open	–	153 metachronous	9 bilateral RN 97 RN/PN 5 bilateral PN	Not reported	Open	73 months	The CSS is better in SBRM than in metachronous RCC
Amano 2010 [22]	Japan	Retrospective	31	22 PN/RN, 8 Bilateral PN 1 bilateral RN	Open	1 RN/RN and 9 PN/RN with one stage, 13/PN/RN and 8 PN/PN with two stages	22	1 bilateral RN, 17 RN/PN 1 PN/RN, 1 bilateral PN, 2 RN/ surveillance	Not reported	Open	58 months	Prognosis of SBRM and unilateral RCC is similar. Metachronous bilateral RCC is associated with higher distant metastasis and mortality rate than SBRM
Berczi 2016 [23]	Hungary	Retrospective	36	17 RN/PN 14 PN/RN 3 bilateral PN	Open	Two stages	24 metachronous	RN + PN	Not reported	Open	57 months	In SBRM PN/PN and PN/RN guaranteed similar oncological and functional outcomes than RN/RN. Tumor progression and cancer-specific mortality were higher in metachronous group
Qi 2017 [24]	China	Retrospective	88	40 bilateral PN 30 RN/PN 18 unilateral surgery	Open	Two stages	60 metachronous	Not reported	Two stages	60 metachronous	76 months	The prognosis in patients with metachronous and synchronous tumors is similar. The OS between patients with bilateral RCC is compatible with unilateral RCC
Kim 2021 [25]	South Korea	Retrospective	44	Unspecified bilateral PN and PN/RN	Open, laparoscopic, or Robot-assisted	Two stages	45 metachronous	Unspecified RN/PN	Not reported	Open, laparoscopic, or Robot-assisted	65 months	No significant difference in eGFR decline in SBRM group compared to unilateral RCC. No perioperative outcomes between SBRM and metachronous lesions
Packiam 2020 [26]	USA	Retrospective	77	Bilateral PN	75 open and 2 minimally invasive	One stage	30	Bilateral PN	Two stages	24 Open, 1 minimally invasive, 5 open/minimally invasive	111 months	No significant differences in oncological outcomes between the simultaneous and staged surgeries
Di Maida 2022 [27]	Italy	Retrospective	17	15 bilateral PN 1 PN/RN 1 RN/RN	11 bilateral robot-assisted, 6 bilateral open	One stage	24	22 bilateral PN 2 PN/RN	Two stages	15 bilateral robot-assisted 2 robotic/open 7 bilateral open	42 months	There were no significant differences between simultaneous or staged bilateral surgeries for SBRM in terms of LRFS and CSS
Pahernik 2007 [28]	Germany	Retrospective	22	Bilateral PN	Open	Two stages	28	RN/PN	Two stages	Open	57 months	Whenever feasible, bilateral PN is the strategy for SBRM, to guarantee an optimal preservation of renal function
Simmons 2010 [29]	USA	Retrospective	134	Bilateral PN	Open or laparoscopic	Two stages	86 RN + PN	60 PN/RN 26 RN/PN	Two stages	Open or laparoscopic	66 months	Double PN should be preferred to preserve the renal function. The 5- and 10-year OS and CSS in patients with SBRM are similar to those with unilateral RCC
Wang 2016 [30]	China	Retrospective	34	Bilateral PN	Laparoscopic	Two stages	24	10 PN/RN 12 RN/PN	Two stages	Laparoscopic	43 months	Two-stage bilateral PN guaranteed optimal preservation of renal function with satisfactory oncological outcomes. The OS and RFS in patients with SBRM is similar with those with unilateral RCC
Ching 2011 [31]	USA	Retrospective	92	Bilateral PN	Open	Two stages	22	Bilateral PN	Two stages	Laparoscopic	66 months	OS of laparoscopic bilateral PN was higher than open approach, while similar results for CSS occurred. Laparoscopic approach was associated with a larger percent decrease in eGFR
Hillyer 2011 [32]	USA	Retrospective	9	Bilateral PN	Robot-assisted	Two stages	17	Bilateral PN	Two stages	Laparoscopic	8 months	RPN guaranteed adequate oncological radicality, with shorter ischemia time and higher renal function preservation than laparoscopic approach

*RN* radical nephrectomy, *PN* partial nephrectomy, *RCC* renal cell cancer, *SBRM* synchronous bilateral renal masses, *OS* overall survival, *CSS* cancer-specific survival, *LRFS* local recurrence free survival

## Discussion

### Results from case-series studies

Eleven case series analyzed patients with SBRM, reporting on varying surgical strategy, functional, and oncological data (Table 1). The management of SBRM is still challenging and different strategies can be considered, namely, PN or RN; open, laparoscopic, or robot-assisted approaches; single or staged procedures; surgery or ablative techniques. The decision is mainly based on surgeon preference, patient’s comorbidity, and tumor characteristic. However, the main goal of treatment is the complete resection of all tumors and the preservation of adequate renal function [33].

Grimaldi et al. [9] evaluated 29 patients with synchronous or metachronous tumors. Twenty-five patients underwent PN and contralateral RN, while the remaining 4 cases had bilateral. Four patients developed metastases, and one patient had a local recurrence after a median follow-up of 52 months. The 5-year overall survival (OS) and CSS were 84.5 and 93.3%, respectively.

Whenever feasible, NSS is also recommended for completely endophytic SRM, given the maintenance of oncological radicality, minimizing the excision of healthy parenchyma [34]. In a retrospective study of 33 patients with central tumors [10], 7 had SBRM. Among the latter, 2 patients underwent staged bilateral PN, while RN followed by PN was performed in the remaining 5 cases. Two patients suffered from a urinary fistula requiring a ureteral stent, while the others showed optimal renal function preservation.

In a study by Blute et al. [11], surgical experience and extended survival outcomes were assessed in 94 patients presenting with sporadic SBRM. Patients were treated with RN + contralateral PN (69%), bilateral PN (26%), or bilateral RN (5%), with most cases (70%) operated on a single session. Among 85 patients with RCC histology, the reported 5- and 10-year OS, CSS, metastasis-free survival (MFS), and local recurrence-free survival (RFS) were 67 and 44%, 81 and 59%, 73 and 66%, and 96 and 93%, respectively. Patients with bilateral RCC (*n* = 71) showed lower 5- and 10-year CSS than those with unilateral RCC (*n* = 14) (79 and 55% vs 91 and 91%, respectively), but this result was not statistically significant. Fuhrman grade 3 disease was associated with metastases, and a significant difference in MFS and CSS was based on the presence of pT3 tumor (*p* < 0.001) but not for local RFS. On multivariable analysis, RCC grade was related to MFS and RCC size with CSS. Type of surgery (i.e., RN + PN vs bilateral PN vs bilateral RN) significantly affected CSS and MFS, yet not local RFS. There was no difference in survival between tumor enucleation and extended, reinforcing the concept that resecting healthy parenchyma around the tumor has no impact on oncological outcomes.

Another study by the same group [12] evaluated 44 patients with sporadic subtype concordant SBRM. The authors evaluated early complications, long-term renal function, and survival comparing those patients with 1714 patients with unilateral RCC. In addition, they assessed the difference in same-sitting vs staged surgery. Pathology findings in patients with SBRM were similar to those patients with unilateral RCC, while the incidence of multifocality in a kidney was larger in patients with SBRM. After controlling for covariates such as RCC subtype, grade, size, and stage, MFS and CSS rates were comparable to unilateral disease, but SBRM patients were more likely to experience local recurrence. Same-sitting bilateral surgery was performed in 84% of patients.

Booth et al. [13] reported their experience of sporadic SBRM treatment, analyzing treatment strategy and type, renal function, and early survival. Among 43 patients, the majority were treated using a staged approach (82%) using bilateral open or laparoscopic PN (LPN) (53.5%). Excluding patients who underwent bilateral RN (*n* = 3), no patients required dialysis after surgery. Considering survival outcomes, 86% of patients showed no evidence of local recurrence after a follow-up of 16 months, one developed metastatic disease, and two died: one of postoperative complications and the other of myocardial infarction.

Woodson et al. [16] explored intermediate oncological and renal functional outcomes of 15 patients surgically treated for sporadic SBRM. All patients underwent staged procedures using different modalities (i.e., PN or RN by open, laparoscopic, or robot-assisted approaches (RAPN)), with bilateral RAPN performed in 53.3% of cases. In 73.3% of patients, the largest tumor was treated first. Although, after the second surgery, there was a significant decrease in renal function compared to pre-operative function, no patients required dialysis. At an average follow-up of 1.7 years, the reported OS and RFS were both 92.8%.

Hu et al. [15] aimed to assess the optimal surgical strategy for sporadic SBRM. In this case series of 32 patients, the surgical type, staging, and sequence were based on the Zhongshan score that includes tumor location and size, and the patient’s performance status (ranked according to ECOG PS Classification). Bilateral single-stage surgery was considered suitable for patients with no comorbidities (*n* = 8), while a staged surgery was 4–8 weeks after treating the first side for larger tumors, complex cases, or poor performance status (*n* = 24,). For bilateral PN (*n* = 15), the operation was performed first on the kidney with a higher Zhongshan score; for patients scheduled to staged PN and RN, RN was conducted first on the side with the largest diameter tumor, while in cases of challenging PN, the latter was done earlier than RN due to the risk of conversion to RN. After a follow-up of 89 months, no patients required dialysis, while one patient presented with metastatic disease and one a local recurrence.

Mason et al. [17] reported perioperative outcomes of 76 patients treated with synchronous bilateral PN. In this case series, 29.7% and 38.2% of patients underwent bilateral and unilateral renal ischemia, respectively. Postoperatively, 21.6% of patients showed complications, with 10.8% of patients experiencing acute renal failure but without the need for renal replacement therapy. At a median follow-up of 3.2 years, 9 cases were metastatic, and eight patients died from RCC.

A peculiar surgical technique was reported in the study by Madi et al. [14], which assessed the use of single-setting bilateral hand-assisted LPN in 3 patients with exophytic bilateral smaller than 4 cm. All procedures were performed successfully, and with no conversion to open surgery or intraoperative complications. Furthermore, no positive surgical margins (PSM) were found, and no local recurrence was detected at a mean follow-up of 51 months.

Recently, few studies have suggested the safety and feasibility of simultaneous bilateral RAPN.

Otoshi et al. [18] reported their experience of same-sitting RAPN in 8 patients with SBRM. In this pilot study, no PSM occurred, and no local recurrence or metastases emerged at the follow-up. Moreover, only one patient developed acute kidney injury without the need for dialysis.

Similarly, Gallo et al. [19] evaluated perioperative and functional outcomes of simultaneous RAPN for non-familiar SBRM. The complication rate was 25.9%, mainly Clavien grade II and only one Clavien grade III (i.e., urinary leakage with perirenal urinoma). The PSM rate was 3.7%, and the RFS was 100% at a median follow-up of 30 months.

### Comparison between synchronous and metachronous tumors

A considerable discriminant in bilateral renal tumors is their presentation, which schematically differentiates into synchronous and metachronous. Evidence from the comparative studies included in our review highlights remarkable findings for differences in the biological behavior between those two types of presentation, especially regarding histologic subtypes, prognosis, and impact on renal function.

In a prospective study by Novick et al. [20], 28 patients with SBRM and 28 with metachronous tumors underwent either staged bilateral PN or RN followed by contralateral PN. Tumor recurrence occurred with a higher percentage in metachronous tumors (46.4% vs 25%,), while their 5-year survival rate was lower (52% vs 73%). The authors concluded that PN for SBRM should be always performed to improve the long-term functional outcomes and the OS.

The largest series was reported by Boorjian et al. [21] at the Mayo Clinic between 1970 and 2003. This cohort included 148 cases of synchronous and 162 cases of metachronous RCC. Metachronous tumors showed a greater pathological concordance than synchronous ones, with 87.7% of metachronous tumors having bilateral RCC compared to 69.2% of synchronous tumors. A longer interval between tumor presentation in metachronous RCC was found to be associated with a better prognosis. Conversely, SBRM showed a comparable survival to unilateral RCC according to a precedent study from the same group [12].

A study by Amano et al. [22] reported similar pathology findings. Interestingly, a higher incidence of concomitant metastatic disease at the time of the second tumor presentation (50% for metachronous vs 13% for synchronous tumors) suggested that metachronous contralateral tumors could be considered as a metastasis of the original tumors.

Qi et al. [24] compared the prognosis of patients treated with bilateral surgery vs unilateral surgery vs no surgery. They reported a higher 5-year CSS in patients with bilateral surgery (93.6%) compared with patients with unilateral (81.5%) and no surgery (0%). The authors demonstrated that OS was significantly better for metachronous non-metastatic RCC (more than 80% at 5 years and 70% at 10-year follow-up) compared to unilateral RCC associated with metastatic disease, suggesting a different biologic behavior between bilateral renal cancer and metastatic disease. Moreover, the majority of metachronous RCC occurred without metastatic disease (81%), while no metastatic disease case was reported at the diagnosis of SBRM.

Evaluating the postoperative loss of renal function, Kim et al. [25] compared the outcomes of bilateral surgery for 44 synchronous and 45 metachronous RCCs. No significant differences in variables among the two groups emerged. Nevertheless, on multivariate analysis, for the prediction of, metachronous RCC was a predictor factor of de novo CKD (HR: 2.682, 95%CI 1.032–6.973, *p* = 0.043).

In summary, SBRM is associated with a more favorable prognosis compared to metachronous tumors. Patients with SBRM exhibit higher OS and CSS rates, along with a lower incidence of distant metastasis. Moreover, no difference in functional outcomes occurs in the surgical management of these tumors.

### Same-sitting vs staged procedures

The surgical treatment of SBRM can be performed in a single session or a staged fashion. No absolute consensus exists on this topic, and current guidelines still lack an optimal surgical approach [35, 36]. Same-sitting bilateral NSS would be the ideal setting to avoid two anesthesia and save costs, although postoperative complications may occur, such as bilateral operative trauma, and acute renal insufficiency. On the other hand, a staged procedure allowed scope to alter the treatment strategy for the second renal lesion based on the pathological findings and outcomes of the first surgery. Given the higher frequency of disease progression in a high-stage tumor, the resection of the larger tumor also provides more histopathological-related information for better planning of the staged surgery [30]. Removing the larger mass first would offer the possibility of reducing the chance of dissemination, and the contralateral kidney could function as a backup instead of being traumatized during the operation, minimizing the chance of dialysis, while PN of the smaller mass, at first, would allow a lower risk of acute renal failure in the second surgery [37].

Mason et al. [17] reported the results of 76 patients treated with same-sitting bilateral PN. Eight cases (10.8%) experienced postoperative acute renal failure with a glomerular filtration rate (GFR) decrease after surgery of 19 mL/min/1.73m^2^, although no patients required postoperative dialysis.

Packiam et al. [26] compared 77 patients undergoing same-sitting bilateral PN vs 30 patients who had staged PN. Compared to staged PN, same-sitting PN demonstrated a lower eGFR reduction at 3 months ( – 6% vs  – 24%; *p* = 0.015) and 12-month post-surgery ( – 4% vs  – 22%; *p* < 0.001). Moreover, the same-sitting approach showed a lower pooled length of stay (6 vs 8 days; *p* < 0.001), urine leak rate (3% vs 17%; *p* = 0.018), and high-grade complications rate (8% vs 23%; *p* = 0.044), confirming that bilateral single stage can be considered safe [26].

Similarly, Di Maida et al. [27], comparing 17 and 24 patients, respectively, treated with a one and a two-stage strategy, reported a significantly higher cumulative operative time (310 vs 240 min; *p* = 0.01), warm ischemia time (18 vs 10 min; *p* = 0.03), and length of stay (10 vs 6 d; *p* = 0.01) for patients receiving the two-stage surgery. However, no significant differences emerged in median eGFR variation from baseline at 3 months and last follow-up, as well as in RFS between the two groups.

Summarizing, the same-sitting approach for SBRM yielded comparable outcomes in terms of oncological radicality and CSS. In addition, renal function preservation was found to be comparable between these two techniques.

### Conservative vs radical surgical strategy

Which is the best surgical approach for SBRM remains unclear as long as oncological principles cannot be compromised vis-a-vis the risk of developing or worsening CKD with subsequent worsening of quality of life [38]; hence, opting for either a bilateral NSS or unilateral RN with contralateral NSS are the only choices available.

Evaluating the study by Kim et al. [25], 44 and 45 patients underwent, respectively, bilateral PN and RN + PN. The former appeared to have less impact on GFR, with the mean postoperative value of 79.4 ml/minute/1.73 m^2^ compared to 61.2 when RN followed by PN was the treatment and 41.4 when PN followed by RN performed. Therefore, surgery sequence was a significative and independent predictor of this study.

In a retrospective study recording data from 57 patients [28], 22 bilateral PN and 28 PN followed by RN were performed. The serum creatinine level was significantly different between the two groups, with bilateral NSS showing a lower level than NSS plus RN. Specifically, the serum creatinine level at the latest follow-up was 1.18 mg/dL for patients after bilateral NSS and 1.40 mg/dL after unilateral NSS and contralateral RN (*p* < 0.05).

Simmons et al. [29] retrospectively analyzed oncological and functional outcomes in 220 patients, whereby 134 patients underwent sequential bilateral PN, 60 had PN followed by RN, and 26 had RN followed by PN, to understand how surgical approaches impact renal function. Comparing the surgical management, a decrease in GFR patients with preoperative stage III CKD was reported in 12%, 43% and 53% of cases in PN-PN, PN-RN and RN-PN groups, respectively. The authors affirmed that patients treated with sequential surgery have 5- and 10-year oncological outcomes comparable to unilateral kidney cancer, with a 10-year CSS of 96%. According to Kaplan–Meier analysis, tumor size greater than 7 was correlated with decreased OS (*p* = 0.003) but not with CSS (*p* = 0.14). Therefore, NSS should be conducted for all amenable bilateral kidney masses due to the negative impact of renal function decline on OS.

Wang et al. [30] reported a retrospective study on 60 patients with sporadic SBRM who underwent retroperitoneoscopic treatment. Of the 56 staged surgeries with the kidney having tumors of a higher PADUA score operated upon first, 34 underwent bilateral PN, 12 underwent RN followed by PN, and 10 underwent PN followed by RN: the final GFR was 71, 63, and 59 mL/min/1.73 m2 in, respectively. Therefore, the preservation of renal function resulted better in the former (*p* = 0.04), while no difference in the order of RN and PN was reported (*p* = 0.79). They reported that the key findings were that, with the advent of the laparoscopic approach, bilateral PN was superior in renal functional preservation with equivalent oncological results and reduced the high risk of postoperative renal dysfunction without any added morbidity of two surgeries.

The above studies reaffirm that a staged minimally invasive approach to managing the kidney with the higher volume tumor burden by either RN or PN as per oncological principles in the first surgery can help mitigate the chance of acute kidney injury and its related consequences. Considering that CSS and OS are similar in SBRM to that unilateral tumors, surgical intervention in experienced centers should be offered.

In brief, NSS is preferable over radical surgery even for SBRM. The survival rates and recurrence rates are similar, but at the same time, the preservation of healthy parenchyma allows for better renal function.

### The optimal surgical approach for bilateral renal masses

Bilateral surgery can be performed with every approach, depending on the surgeon’s expertise and availability [39, 40].

Gill et al. reported that laparoscopic PN offers advantages in terms of operative time decrease, less blood loss and ischemia time, and fewer complications compared to open surgery, with both equal renal functional and oncological outcomes, despite the increasing surgical complexity [41].

On the contrary, Ching et al. [31], comparing data and outcomes of 92 patients undergoing bilateral open PN and 22 patients treated with bilateral laparoscopic PN, reported a significantly higher percentage decrease of GFR after laparoscopic compared to open surgery (38% vs 27%, *p* = 0.03). There was no difference in CSS and RFS rates between the two approaches.

RAPN was reported as a reliable surgical approach to minimize the technical limits linked to LPN, as well as the longer operative time and the risk of CKD, which may occur for simultaneous bilateral PN [42]. RAPN showed to be superior to open and laparoscopic approaches due to a better preservation of renal function, a decrease in intra-operative bleeding, shorter ischemic time and postoperative stay [43, 44].

Otoshi et al. [18], summarizing the results of 8 cases of simultaneous RAPN for SBRM, reported no PSM or local recurrence with only one patient who experienced acute renal failure not requiring dialysis.

Hillyer et al. [32] compared the intra- and postoperative outcomes of bilateral RAPN in 9 patients to bilateral LPN in 16 patients. There was no difference in terms of operative complication rate, although a trend toward a shorter warm ischemia time in the RAPN group (19 vs 37 min; *p* = 0.059) occurred, with a higher postoperative eGFR (68.7 mL/min/1.73 m2 vs 26.9 mL/min/1.73 m2, *p* = 0.004). The authors concluded that RAPN is an effective and safe procedure for bilateral PN.

In summary, open, laparoscopic, and robot-assisted approaches have shown similar results in terms of oncological outcomes. However, robot-assisted surgery has demonstrated a distinct advantage in terms of reducing complications associated with the procedure.

### Limitations

This scoping review has certain limitations. Most of the included studies were retrospective and evaluated small sample sizes, which inevitably introduces low quality of evidence. Therefore, it is crucial to conduct larger and higher quality investigations in the future to better understand the surgical management of SBRMs. Another limitation is the variation in surgeon expertise across the considered studies, which generally influences the chosen approach. Consequently, performing NSS for complex SBRMs may only be recommended for expert surgeons. Similarly, the choice between open or minimally invasive approaches, as well as the timing of the surgery, were be standardized among included studies. Finally, the significant differences in interventions among the included studies prevent a comprehensive analysis of the overall oncological and functional outcomes. Consequently, definitive conclusion cannot be drawn.

## Conclusion

Our review provides valuable insights into the surgical management of SBRM, which showed a better long-term OS and CSS than metachronous tumors. The recommendation for conservative surgery for SBRM relies on the preservation of renal function. Our study also points out that there is currently no oncological advantage of one surgical approach over the others, but RAPN seems to offer better perioperative outcomes in terms of preservation of renal function and early postoperative outcomes. The comparison between same-sitting vis-a-vis staged bilateral NSS is also significant, with similar renal function preservation but better perioperative outcomes for the former.

## Supplementary Information

Below is the link to the electronic supplementary material.

Supplementary file1 (DOCX 45 KB)

## Data Availability

Data will be provided by the corresponding author upon a reasonable request.
