# Supplementary material for: The current evidence on surgical management for synchronous bilateral renal tumors: results from a scoping review
Source: World J Urol. 2023 Jul 5;41(8):2107–18. doi: 10.1007/s00345-023-04503-y (PMC10415478; doi:10.1007/s00345-023-04503-y)
Supplement: Supplementary file 1 — Supplementary file1 (DOCX 45 KB) [file 345_2023_4503_MOESM1_ESM.docx]

**Figure 1:** *PRISMA 2009 flow diagram*

**Identification of studies via databases and registers**

Records identified from databases (n = 2238)

- PubMed (n = 684)
- Embase (n = 1389)
- Scopus (n = 165)

Records removed *before screening*:

Duplicate records removed (n = 712)

**Identification**

Records screened

(n = 1526)

Records excluded

(n = 1453)

Reports sought for retrieval

(n = 73)

Reports not retrieved

(n = 0)

**Screening**

Reports excluded (n = 49):

Meeting abstract (n = 25)

Not in English (n = 16)

Wrong outcomes (n = 5)

Wrong indication = 1)

Wrong study design (n = 1)

Review (n = 1)

Reports assessed for eligibility

(n = 73)

Studies included in review

(n = 24)

**Included**
